# The Mississippi Valley Medical Association

**Published:** 1887-07

**Authors:** 


### THE MISSISSIPPI VALLEY MEDICAL ASSOCIATION.

The forthcoming meeting of the Mississippi Valley Medical Association will be one of great importance to that body. Since the adoption of the code of ethics as part of its organic law, the association has had a most remarkable development. It is intensely loyal to the American Medical Association, but believing that body too large and unwieldy to consitute a good *working* society from a scientific point of view, it has taken to itself the work of establishing in the West and South a society which shall stand upon principles broad and comprehensive, having for its first object a high grade of scientific work from its members.

The chief features of importance in this association, as we understand them, are, *a*, its method of membership. It is a non-delegate body. If the constitution which is to reported at the next meeting is adopted, membership will be limited to those members of the profession of medicine who acknowledge allegiance to the American Medical Association by signing its code of ethics. This requirement we consider simple, and at the same time ample enough to meet all emergencies. *b*. The manner of disposing of its transactions and scientific communications. Believing that the time has passed when it is proper to bury scientific communications in a volume called "Transactions," which appears frequently as late as one year after the meeting, the Mississippi Valley Medical Association lays no restrictions whatever upon contributors except that contributions presented to the society shall not appear in any but the regular medical journals.

With these two important features in its constitution, we cannot but believe that the association will become one of the foremost in the country.

But there are other things to which this society might well turn its attention. We believe that, instead of a long array of papers upon hackneyed subjects, this association should give its attention to some of the live questions of the day. It might with much profit to itself and others devote considerable time to the discussion of such subjects as "The present condition ' our public schools and its effect upon the health of the the young," "Manual training," "The organization of the medical profession," and many others of equal importance.

